# Supplementary material for: Are carbon emissions trading and green financial instruments synergistic? -Comprehensive quantitative research based on content analysis
Source: PLoS One. 2024 Mar 7;19(3):e0298601. doi: 10.1371/journal.pone.0298601 (PMC10919601; doi:10.1371/journal.pone.0298601)
Supplement: S1 Data — (ZIP) [file pone.0298601.s001.zip › Data/地级市Z_F工作报告.zip!/╜¡╦╒/╠⌐╓▌╩╨/└┤╫╘╣1⁄2╓┌║┼í╛┬φ┐╦╩2╛▌═°í┐_2005.docx]

各位代表：

现在，我代表市人民政府向大会作工作报告，请予审议，并请市政协委员和其他列席人员提出意见。

一、2004年工作回顾

过去的一年，是我市国民经济加快发展、改革开放全力推进、人民群众受益较多的一年。在中共泰州市委领导下，全市人民认真贯彻党的十六大和十六届三中、四中全会精神，紧紧围绕力争“五年总量翻番、八年全面小康”奋斗目标，树立和落实科学发展观，自觉服从和主动适应宏观调控，努力推进经济社会全面协调可持续发展，圆满完成了市二届人大二次会议确定的各项目标任务。

经济持续快速增长，综合实力进一步增强。扎实推进结构调整，积极化解紧运行中的突出矛盾和问题，促进经济又快又好发展。全市实现地区生产总值705.2亿元，人均14690元，可比分别增长14.7%和14.6%，增速均为建市以来最好水平。其中，第一产业增加值82.7亿元，增长4.5%；第二产业增加值380.5亿元，增长18.7%；服务业增加值242亿元，增长12.5%。抓好粮食生产，保障和促进农业结构调整。深化农村税费改革，认真落实扶持粮食生产等一系列政策，调动了农民生产积极性，全年粮食增产27.9万吨，实现了粮食丰收、农业增效、农民增收的目标。重点打造弱筋小麦产业，种植面积扩大到35万亩。有效防治禽流感，多种经营稳定增长。出台扶持农业产业化龙头企业加快发展的意见，龙头企业对基地和农民的带动作用进一步增强。加强对外招商，农业利用“三资”超过15亿元。市农业开发区建设起步良好，行政体制和产业经营的主要架构初步形成。基本建成市农产品质量检测中心和重大动物疫病监控体系。认真实施“第一方略”，推动工业提速增效。规模以上工业实现销售收入879.9亿元、增加值282.1亿元、利税86.6亿元、利润43亿元，分别增长33.3%、23.2%、26.2%和26.8%，其中增加值增幅列全省第4位。着力推进“2211”工程，30强企业发展势头良好。工业技改投入增势强劲，全年完成153.6亿元，增长49.5%，实现了两年总量翻一番。170个重点技改项目全部开工建设，其中81个项目已竣工投产。新培植市级重点新产品210个，其中71个当年达产达效。强化市场开拓和结构调整，加快建筑业发展。全年完成建筑业施工总产值302亿元，增加值61亿元，分别增长25.2%和12.9%。江苏一建进入全国承包商60强。突出抓好重点行业、重点项目，服务业增长速度进一步提升。全年社会消费品零售总额200.7亿元，增长15.5%，比上年提高3.1个百分点。房地产业、信息服务业、社区服务业和旅游业成为新的增长点。25个服务业重点项目建设加快推进。财税金融工作取得新成绩。完成财政收入86.5亿元，增长35%，其中地方一般预算收入33.9亿元，增长39.3%，增幅列全省第3位。年末各项存款余额712亿元，各项贷款余额403亿元，分别比年初增长17.1%和18.5%。

沿江开发进程加快，对外开放取得新成绩。建立健全沿江开发体制、机制，加大推进力度。沿江开发总体规划正式实施，相关专项规划、分区规划编制完成。加强岸线开发利用管理，促进集约开发、持续开发。突出抓好沿江重大项目建设实施，泰州电厂、过江通道项目前期工作进展顺利，泰州港集装箱码头、陵光重交沥青—燃料油一期等重点项目如期竣工，沿江高等级公路、扬子江药业城、新浦化工二期等重点项目加快推进。促进跨江联动开发，江阴经济开发区靖江园区各项基础设施建设进展顺利。对外贸易保持快速增长，完成自营出口额9.54亿美元，增长50.4%，增幅列全省第4位。进一步加大招商引资力度，赴日本、韩国和台湾、香港、厦门等地成功举办了招商活动，组团参加了一系列省以上大型经贸洽谈活动。深入开展“五比五赛”，促进了省级经济开发区和海陵工业园、高港科创园等重点园区的建设和招商。全年协议利用外资7.93亿美元，增长65.4%；实际利用外资4.17亿美元。对外经济技术合作势头良好，新签对外承包劳务合同额1.34亿美元，完成营业额1.43亿美元，分别增长15%和66.5%。海关、国检、海事等口岸查验机构为开放型经济发展作出了新贡献。友好城市间的交流合作取得新进展。

各项改革继续推进，民营经济快速发展。认真抓好市（区）属工贸企业“三置换一保障”改革扫尾工作，全市789家工贸企业中782家已完成改制，改制面达99％。交通、建筑等行业企业改革着手推进。抓好经营性服务类事业单位改革，全市已有261家事业单位完成改制任务，市属首批经营性服务类事业单位改制基本结束。制定出台《关于鼓励民营经济加快发展的若干意见》，加大信用担保、创业培训、信息咨询、权益保护等服务和支持力度，鼓励和促进全民创业。全市新增私营企业3846家，新增个体工商户3.3万户，注册资本增加62.6亿元。私营个体经济实现增加值293亿元，占全市总量的41.5%；上缴税金16.9亿元，比上年增长29.9％。贯彻实施《行政许可法》，深入开展行政许可项目清理和实施主体认定工作，停止执行20个行政许可项目，向社会首批公布了268个行政许可项目和59家行政许可实施主体。市行政服务中心运转正常。部门预算全面推开，市本级国库集中收付制度基本建立。

城镇建设步伐加快，基础设施建设取得新进展。按照“三年形成大城市框架，五年达到大城市标准”的目标，编制了中心城市建设三年实施纲要。以年度“十大工程”为重点，扎实推进新区建设和老城改造，中心城市建设投资达到50亿元。凤凰东路、金港北路等主次干道新建和改造工程如期竣工，九大防洪工程有序推进，长江水郊区达镇管道建设完成，海陵城区燃气管网实现全方位覆盖，市第一污水处理厂投入运行，市垃圾填埋场、滨江污水处理厂开工建设。环城河风景区一期和泰山公园改扩建工程即将完成。老城区一批后街背巷和低洼地得到改造，南园小区整治结束。市区新增绿地面积220万平方米，新建绿化小游园7个，住宅竣工面积40万平方米。不断完善城市项目建设“五大机制”，城市经营水平进一步提高。加强城市管理工作，建设施工环境得到整治，“畅通工程”通过省验收。市城市管理行政执法局成立。各辖市加大城市建设投入，城区面貌发生了较大变化。重点中心镇建设步伐加快，村镇详规与村庄规划试点进展顺利。进一步完善城乡基础设施。334省道泰兴绕城段、328国道姜堰绕城段、宁盐公路姜堰段、江平公路江都界至高港观音堂段等道路建成通车，宁启铁路泰州站加快建设。新建、改造农村公路1411公里。泰东河拓浚工程完成年度建设任务，里下河地区灾后重建应急工程竣工。农村水利建设在农民负担政策调整后继续保持良好的势头。500千伏泰兴至斗山变线路工程等一批重点电力设施建成投运。邮政、通信等基础设施建设也取得了新进展。全年全社会固定资产投资306.2亿元，增长32.8%。

社会事业协调发展，人民生活进一步改善。继续实施“科教兴市”战略，成功举办“企业院校行”活动，全年组织实施省以上火炬、星火计划项目45项，新认定省级高新技术企业25家、高新技术产品51个，我市被评为全省科技进步先进市。加强人才高地建设，引进各类人才11629人。高等教育发展取得新突破，南京理工大学泰州科技学院、南京师范大学泰州学院挂牌成立并招收本科学生1096人，高教园区建设前期工作全面展开。基础教育教学水平不断提高，农村中小学布局调整提前通过省级验收。职业教育省级示范专业创建工作取得新成绩。市莲花学校新校区一期工程开工建设。出台《泰州市2004—2010年文化建设规划纲要》。成功举办市首届学术年会和纪念海军诞生55周年、梅兰芳诞辰110周年、央视空中剧院江苏泰州行等文化活动，提升了泰州知名度和影响力。城隍庙修缮工程竣工，光孝寺扩建工程进展顺利。《泰州年鉴》（2004年卷）编辑出版。全市有线广播电视通村率达98%，城乡综合入户率达51.4%。泰州有线数字电视平台建成并投入试运行。公共卫生体系建设得到加强。市人民医院南院门急诊综合楼、市口腔医院综合楼竣工，高港区人民医院迁建工程顺利实施，市卫生监督所综合楼和市传染病医院一期工程开工建设。市疾病预防控制中心实验室通过国家认可。市体育中心一期工程交付使用。成功举办市第二届运动会。巩固计划生育成果，人口出生率稳定在较低水平。耕地保护和土地复垦整理力度加大，土地市场秩序治理整顿取得成效。认真做好征地补偿拖欠清理工作，积极推进被征地农民生活保障制度。扎实抓好违法排污企业整治、放射源清查工作，全市生态环境质量综合评价指数在全省名列前茅。第一次全国经济普查年度工作完成。气象、防震、史志档案、民族宗教、妇女儿童、老龄、残疾人等工作得到加强，质监、药监、工商管理和对台、外事、侨务在服务地方经济发展中取得新成绩。城乡居民收入较大幅度提高，城镇居民人均可支配收入9695元，农民人均纯收入4650元，分别增长13.8%和14%。年末城乡居民储蓄存款余额467.3亿元，比年初增长16.2%。加强就业再就业工作，全市城镇新增就业人员2.6万人，下岗失业人员再就业1.99万人，登记失业率为3.8%。市政府被国务院授予“全国再就业先进单位”。全面落实“两个确保”和城镇居民最低生活保障制度，全市退休人员医疗保险实现全覆盖，“金保工程”基本完成。强化农村劳务培训和输出服务，全市新转移农村劳动力8.3万人。为市区人民办的10件实事基本完成，农村“5+1” 实事工程达到预定进度。市区新增就业人员6306人，救助特困家庭1102户，5万平方米低价房主体工程封顶。农村低保实现全覆盖，以大病统筹为主的农村新型合作医疗保险参保率达70.5%，农村自来水受益人口新增19.3万人，农户草危房改造完成3553户。

精神文明创建活动深入开展，民主法制建设继续加强。扎实推进“五城同创”，在成功创建全国双拥模范城的基础上，文明城市、卫生城市、园林城市和环保模范城市创建工作取得阶段性成效。深入开展“富民为先、创业为本”主题宣传活动，激发了广大群众自主创业的热情。在全市青少年中广泛开展了“弘扬‘三创’精神，争当新一代泰州人”系列教育活动，未成年人思想道德建设得到加强。组织开展文明社区（小区）、文明行业（单位）、文明村镇、文明家庭创建评比活动，推动创建工作向非公经济单位和工业园区拓展。加强国防教育，兵员征集、民兵预备役和双拥工作取得新成绩。重视人民防空事业，人防工程建设取得新进展。民主法制建设继续推进。自觉接受市人大的法律监督、工作监督和市政协的民主监督，重视和发挥各民主党派、无党派人士、工商联及群众团体的参政议政作用。建立健全联系市人大代表和市政协委员工作制度，认真办理人大代表建议和政协委员提案，满意（含基本满意）率为98.7%。全市第七届村委会换届选举工作进展顺利。编制《建设法治泰州实施纲要》。继续抓好“四五”普法教育，全民法制意识进一步提高。法律援助体系不断完善。信访工作进一步加强，建立了集中处理群体性事件联席会议制度，妥善解决了大量信访问题。民间矛盾纠纷“大调解”机制初步建立。“平安泰州”创建工作扎实推进，社会治安保持稳定，四市两区建成全省社会治安安全市（区）。整顿和规范市场经济秩序，“诚信泰州”建设取得新成效。按照“建设学习型、服务型、实干型、廉洁型政府”的要求，进一步加强政府自身建设。积极打造学习型机关，提高公务员队伍素质。开展基层站、所作风建设活动，加强行政效能监察，深化经济发展软环境建设。认真办好市长公开电话和市长信箱，了解社情民意。全面启动“221工程”，30个薄弱乡镇经济实现了稳步增长。机关干部结对帮扶城市贫困户活动取得新成效。在全省率先出台政府信息公开办法，政务公开工作取得新进展。电子政务建设逐步推开。严格执行廉政责任制，领导干部廉洁自律工作进一步加强，征地拆迁中的不正之风和教育乱收费得到较好治理和纠正，查处违法违纪案件取得新进展。

各位代表，过去一年政府各项工作取得的成绩，是在中共泰州市委的正确领导下，在市人大、市政协的监督和支持下，全市人民团结拼搏、共同努力的结果。在此，我代表市人民政府，向全市各行各业的广大干部群众，向各民主党派、工商联和无党派爱国人士，向中央和省驻泰机构以及驻泰部队指战员、武警官兵和公安干警，向所有关心和支持泰州现代化建设的海内外朋友们，表示衷心的感谢！

过去的一年，全市经济和社会发展中还存在不少问题。一是农民增收的长效机制有待逐步建立。农业综合生产能力还没有实质性提高，农资价格涨幅较大，农民持续增收困难。二是服务业发展仍相对滞后。经济增长主要还是依靠投资拉动和工业支撑，服务业发展的速度和水平有待进一步提升。三是利用外资的规模还不够大。虽然利用外资增长速度较快，但与周边地区相比总量仍有一定差距，上亿美元的大项目仍然偏少。四是社会稳定工作压力较大。就业再就业形势仍较严峻，各种可能诱发不稳定的因素增多，给维护社会稳定带来新挑战。五是政府自身建设还存在一些薄弱环节。政府职能转变还不适应发展的要求，依法行政能力需要进一步提高，等等。我们一定高度重视这些问题，采取有效措施，切实加以解决。

二、2005年工作任务

各位代表，今年是全面贯彻党的十六届四中全会精神、进一步落实科学发展观的重要一年，是推进力争“五年总量翻番、八年全面小康”进程的关键之年，也是实施“十五”计划的最后一年。综观整个发展形势，我们面临着较多的发展机遇和有利条件。全球经济复苏态势趋于稳定，投资和贸易将更加活跃；我国经济景气上升周期延长，国内需求继续保持旺盛态势；长三角地区仍是国际资本和产业转移的重点地区，发展活力可望进一步增强；我市近年来推进大改革、大开放，持续大投入、大建设，其效应将进一步显现，经济快速增长的基础比较扎实；尤为重要的是，全市广大民众投资、创业的热情不断高涨，必将为加快发展注入强大动力。同时，我们也应看到，世界经济环境变数较大，国内宏观调控继续加强，重要生产要素供给仍然较紧，将给经济社会发展带来一定影响。我们要审时度势、抢抓机遇，积极应对、克难求进，努力实现经济社会又快又好发展。

今年政府工作的总体要求是：深入贯彻党的十六大和十六届三中、四中全会精神，认真落实市委二届八次全会的决策部署，以“三个代表”重要思想和科学发展观统揽全局，加快富民步伐，加快改革开放，加快经济结构调整，加快城市化进程，加快社会事业进步，协同推进“三个文明”建设，着力构建和谐社会，促进全市经济社会全面协调可持续发展。

今年全市国民经济和社会发展主要调控目标初步安排为：全市生产总值增长12%以上，其中第一产业、第二产业、服务业增加值分别增长4%、15%和11.5%以上；财政收入增长20%，其中地方一般预算收入增长18％；全社会固定资产投资增长20%；社会消费品零售总额增长12%；自营出口额增长18%；实际利用外资6亿美元；城镇居民人均可支配收入增长10%，农民人均纯收入增长8%；城镇登记失业率控制在4%以内；人口自然增长率控制在4‰以内。

按照今年政府工作的总体要求，围绕实现全市国民经济和社会发展主要调控目标，我们将着重做好五个方面的工作：

1、坚持富民优先，努力提高人民生活水平。始终不渝地促进民富，改善民生，保障民安，让人民群众从改革发展中得到更多实惠。

大力发展民营经济。围绕形成“铺天盖地”发展之势，广泛开展“创业之星”争创活动，激发全民创业热情；强化创业指导、培训和服务，认真落实鼓励民营经济加快发展的政策意见，支持民众创业实践。围绕形成“顶天立地”的规模企业群体，引导民营企业加快机制创新、技术创新、管理创新，不断增强市场竞争力；支持100家规模型、科技型、外向型、再就业型民营企业做大做强，培育一批全省乃至全国的“单打冠军”。围绕形成“组团扎堆”的特色经济板块，按照“产业集聚、区域集中、开发集约、能量集合”思路，推动民营企业向工业规划发展区集结，培育一批企业集群和特色产业板块。

扩大农村劳务输出。实施“百万农民培训工程”，着力打造具有地方特色的劳务品牌；强化劳务交流协作、输出基地建设和骨干中介组织培育，进一步提高组织程度；完善市县乡劳务信息网络，实现输出信息的实时提供；积极提供法律服务和援助，保障外出劳务人员合法权益。

加强就业和社会保障工作。把就业作为经济社会发展的重要目标，千方百计扩大就业空间，全年城镇新增就业人员2.2万人。注重发展就业容量大、吸纳能力强的劳动密集型产业和社区服务业，注重发挥民营经济、中小企业吸纳就业的作用；继续实施“153”创业培训工程，指导和引导自谋职业、自主创业；健全完善再就业援助制度，帮助“4050”失业人员、家庭双下岗人员以及特殊困难群体就业再就业；认真落实再就业扶持政策，扩大再就业小额贷款担保的受益面。进一步完善社会保障体系。继续巩固“两个确保”和城乡低保，适时提高低保标准；制定出台对新办非公有制企业和灵活从业人员、困难群体参加社会保险给予补贴的政策，扩大社会保险覆盖面；建立被征地农民社会保障制度，提高农村新型合作医疗保险参保率。健全社会保障基金监管机制，确保基金安全。实行农村部分计划生育家庭奖励扶助制度，建立有利于计划生育的政策体系。继续发展社会救灾、救济、互助、福利等社会保障事业。

坚持为民多办实事。把为民办实事作为政府的重要职责，确保全市农村“5+1”实事工程达到既定进度，继续为市区人民办好10件实事。加快定向销售经济适用房建设，完善土地征用、房屋拆迁程序和补偿机制，维护被征地农民和被拆迁户的切身利益。认真清理拖欠工程款和农民工工资，重视维护企业职工的合法权益。坚持和落实资助家庭经济困难学生入学制度，严厉查处教育乱收费和违规收取进城农民工子女入学费的行为。建立和完善大病救助基金正常增长机制，坚决纠正药品购销、医疗服务中的不正之风。重视解决社会弱势群体和因灾因病致贫家庭的生活困难，继续开展农村扶贫和城市结对帮扶工作。全面取消农业税，认真落实粮食直补、良种补贴和购买农机补贴政策。整顿和规范市场经济秩序，建立食品、药品安全综合整治和长效管理机制，强化物价监控。继续实施“221工程”，扩大挂钩帮扶部门范围，认真落实各项帮扶政策措施，加快黄桥老区和里下河地区经济薄弱乡镇发展。

2、突出沿江开发，增创对外开放优势。紧紧抓住国际资本和产业向长三角地区转移的机遇，把沿江作为扩大对外开放的前沿阵地，促进开放型经济实现新跨越。

着力推进沿江开发。强化开放开发，进一步扩大对外招商，力争引进一批关联度大、带动性强、产业链长的基地型、龙头型、旗舰型项目，加快沿江化工、医药、机电、船舶、新材料等五大特色产业的发展。抓好联动开发，促进江阴经济开发区靖江园区的建设和发展。坚持可持续开发，严格沿江岸线、土地开发利用管理，重视沿江生态建设和环境保护，努力实现资源的集约开发和可持续利用。扎实推进沿江重大项目建设，抓紧在建重大产业项目实施，抓好泰州电厂一期工程、离子膜烧碱、新扬子造船工程等10个第二批重大产业项目的开工建设，做好泰州过江通道前期工作，建成宁启铁路泰州站客站广场和通站道路，加快沿江高等级公路泰州段建设。

强力推进招商引资。把握国际资本和产业转移新趋势，积极利用港台资本、突出招引日韩客商、努力扩大欧美投资，力求在利用外资规模上取得新突破。抢抓服务业扩大对外开放新机遇，在抓好工业和农业、加快推进社会事业和基础设施对外招商的同时，力求服务业引资取得新突破。针对国家宏观调控新形势，更加注重高新技术项目招商，注重资源节约型、能源节约型、环保型项目招商，鼓励“零土地”招商和外商增资扩股。根据产业集群化发展新要求，注重产业链招商和基地型招商，重点招引机电、化工、新材料等产业项目。精心组织好日韩招商会，及早策划好第五届科技经贸洽谈会。进一步提高招商组织程度，强化领导力度、招商队伍、引资责任和投资服务。全年新批超千万美元项目30个以上，争取引进超亿美元项目2—3个。

大力促进园区发展。把园区作为招商引资主阵地、产业集聚主载体、区域发展主板块来培育和建设，突出重点，有序推进，逐步形成布局合理、错位发展、特色鲜明的产业园区群。引导5个省级经济开发区和海陵工业园、高港科创园主攻大项目、大客商，实施大投入、大建设，增强产业集聚功能，发挥招商龙头作用；支持春兰、扬子江、陵光、梅兰、新浦、兴达等重点企业园区依托主导产业，推进配套、协作招商，加快建成集团式、集群式发展的产业集聚区；鼓励重点工业规划发展区走专业化、特色化、集约化发展之路，集结民资、集中民企，加快形成地方特色经济板块。进一步创新园区管理体制和运行机制，深入开展“五比五赛”活动，促进各类园区加快发展。

努力扩大外贸出口。紧紧抓住世界贸易持续增长的机遇，加快转变外贸增长方式，保持外贸出口快速增长。加大市场开拓力度，优化出口产品结构，进一步扩大机电、纺织、服装等优势产品和高新技术产品出口。积极申报国家医药出口基地，促进医药产品出口和产业发展。充分发挥海关保税物流中心作用，形成外贸进出口新平台。加强口岸建设，加大港口投入，打造泰州港品牌，建成泰州边防检查站，畅通快捷出口通道。认真落实出口退税政策，进一步改善外贸出口环境。继续实施“走出去”战略。鼓励企业境外投资，扶持企业开展境外加工贸易；做好“窗口型”外经公司注册登记和外经经营权申报工作，扩大对外工程承包和劳务合作规模。重视和做好外事、侨务和对台工作。

3、优化产业结构，促进经济又快又好发展。突出结构调整主线，着力转变经济增长方式，提高发展的速度、质量和效益。

以提高农民的组织化程度为抓手，加快农业产业化进程。在稳定粮食生产的基础上，推进农业产业发展。在继续重点打造弱筋小麦产业的同时，着力抓好优质稻米、加工型蔬菜、银杏、畜禽、特色水产等优势产业，形成一批具有本地特色的农产品品牌；积极培育各类农产品经营主体和专业合作组织，探索联合分散生产的农民走向市场的途径，实现农业的企业化生产经营；完善和落实相关政策，大力扶持农业产业化龙头企业发展，加快形成龙头企业集群；继续加大力度，改进和加强农业招商引资工作，争取实际利用“三资”18亿元；抓好市农业开发区建设，逐步发挥载体和龙头作用；推动农业服务体系改革，强化农业服务功能，抓好动植物疫病防治，保障农产品生产安全；依法、自愿、有偿地流转农村土地承包权，促进适度规模经营。

以新型工业化为方向，推动工业集约高效发展。继续优化产业结构、增加有效投入、培育规模企业、实施名牌战略、深化企业改革，加快新型工业化进程。大力发展高新技术产业，积极运用高新技术特别是信息技术改造和提升传统产业，重点抓好30个年销售过亿元重点新产品培育和开发，进一步壮大机电、化工、医药、冶金船舶、纺织服装及新材料等六大支柱产业；坚持增加投量与优化投向、加快投速与注重投效的有机统一，注重强化对节地型、节能型、节材型和循环经济型项目的支持，突出抓好40个投入亿元以上重大技改项目开工建设，全年技改投入确保完成200亿元；继续实施“2211”工程，引导30强企业注重内涵发展，转变增长方式，扩大规模、增强实力；继续实施品牌战略，开展争创名牌产品和驰名商标活动，全年新增省级以上名牌产品16个；巩固和扩大市（区）属工贸企业“三置换一保障”改革成果，促进股权合理流动，深化企业内部改革，积极争取上市，加快建立现代企业制度。进一步加大建筑业改革改制、资源整合和市场开拓力度，推动我市由建筑大市向建筑强市迈进。全年建筑业施工总产值、建筑企业增加值均增长15%以上。

以拓展发展空间为关键，大力发展现代服务业。抓住宏观经济政策鼓励扶持服务业发展的有利时机，促进传统服务业改造提升，加快现代服务业发展步伐。加强重点市场、大型超市和特色街区建设，大力发展连锁经营、物流配送等新兴业态，进一步提升商贸流通业发展水平。依托港口、重点开发园区和大型商贸市场，加快综合物流园区和专业物流中心建设，推进现代物流业加快发展。规范房地产业市场，活跃住房二级市场和租赁市场，促进房地产业健康发展。强化政策扶持引导，加快社区服务业发展。优先发展旅游业，抓紧编制全市旅游业发展规划，继续推进环城河风景区和溱湖风景区建设，积极开展争创国家级、省级旅游品牌活动。继续推进保险、信息和中介服务业、文化和体育产业加快发展。进一步规范税收征管，调整优化财政支出结构，增强调节和保障功能。注重化解乡镇财政风险，逐步解决部分乡镇财政运转困难的问题。引导各商业银行调整信贷结构，优化信贷投向，强化对经济发展的信贷支持。

4、统筹城乡建设，进一步改善区域发展环境。加快中心城市、城乡基础设施和生态环境建设，构建城乡联动、跨越发展新平台。

加快中心城市建设。遵循城市建设总体规划，坚持年度建设与三年纲要实施相结合，新区建设与老城改造相结合，完善功能与提升形象相结合，围绕“出精品、出亮点、出形象”，着力推进重大工程、重点地区、重要路段建设，全年中心城市建设投资力争完成60亿元。突出抓好文化中心、泰高路扩建、坡子街商业中心、铁塔广场等十大重点工程，进一步提升城市形象；健全完善决策、融资、实施、偿债、监督五大机制，充分发挥城投公司投融资平台作用，进一步提升城市经营水平；继续推行综合执法，加大建设施工环境专项整治、违章建筑拆除工作力度，进一步提高城市管理绩效。坚持把文化建设渗透到城市规划、建设和管理的全过程，不断提升城市建设品位。按照“一大四中”的城市发展格局，引导各辖市完善城区规划，强化基础设施建设，加强城市管理，进一步提升城市发展水平。全年四市城区建设投资力争完成70亿元。继续抓好重点中心镇规划和建设，促进人口和产业集聚，培育一批戴南、张郭、黄桥式新型城镇。

进一步完善城乡基础设施。按照适度超前、突出重点、城乡一体、配套完善的要求，加快城乡基础设施网络化、现代化进程。年内姜八公路泰兴段、高兴东公路兴化南绕城段建成通车，新建、改造农村公路1300公里。继续抓好防汛抗旱、区域骨干水利工程和农村河道疏浚等农业基础设施建设，启动泰东河姜堰段河道拓浚工程，完成草河、城南河等河道清淤活水。加强城乡电网建设，完成双墩变、田河变建设和顾庄变扩建工程，开工建设泰兴变2号主变和王石、唐子输变电工程。完善信息基础设施，实施电信、移动、联通网络扩容工程。

加强区域生态环境建设。组织实施《泰州生态市建设总体规划》，加快建设“蓝天、碧水、绿色、洁静”的生态城市。严格执行控制大气污染的有关规定，进一步净化城市空气；加强城市污水收集处理，抓好长江、里下河水污染防治；开展全民义务植树活动，建设绿色城市、绿色企业、绿色家园，打造“绿色泰州”；治理城市噪声，提高声环境质量。积极推行清洁生产，推广使用清洁能源，大力发展循环经济。强化环境监控和环保管理，坚决制止高污染项目。开展生态建设争创活动，建成市级以上环境优美乡镇4个、生态村10个。继续实行严格的耕地保护制度，促进可持续发展。

5、注重协调发展，着力推动社会全面进步。在促进经济又快又好发展的同时，重视社会协调发展，维护社会稳定，构建和谐社会。

加快发展各项社会事业。切实加强技术创新体系建设，加快高新技术产品研制和开发，培育高新技术企业，重视知识产权保护，争创全国科技进步先进市。创新人才引进、培育和使用机制，建设人才高地。继续巩固提高“普九”成果，大力发展高中阶段教育和高等教育，重视职业教育和成人教育。全面启动中小学教育信息化“校校通”工程，建成市莲花学校新校区一期工程，加快高教园区建设步伐，扩大南京理工大学泰州科技学院、南京师范大学泰州学院招生规模。促进民办教育规范、健康发展。贯彻《文化建设规划纲要》，启动实施泰州市民族民间文化保护工程。抓好光孝寺续建，修复扬郡试院。继续推进有线广播电视进村入户，完善有线数字电视平台。进一步加强公共卫生体系建设，完成市卫生监督所综合楼建设和市传染病医院一期工程，开工建设市人民医院南院病房楼，组建市急救中心。稳定低生育水平，提高人口素质，进一步提升计划生育科学管理和优质服务水平。实施全民健身计划纲要,发展体育事业。承办好全国十运会泰州赛区比赛。完成第一次全国经济普查年度任务。继续发展史志档案、社会科学、新闻、科普、气象、防震等事业，做好妇女儿童、老龄、残疾人等工作。

加强精神文明建设。扎实推进中心城市“五城同创”，巩固“双拥”创建成果，力争通过全国文明城市创建工作先进市、国家卫生城市、国家环保模范城市、省级园林城市验收。认真抓好“四市联创”，泰兴市同步实现“五城同创”阶段性目标，靖江、姜堰、兴化三市争创省级文明城市。启动“小康示范村”、“现代化示范村”创建活动，提升村级管理水平。加强社会信用体系建设，打造“诚信泰州”。切实做好国防教育、民兵预备役、兵员征集和双拥工作。完成市人防指挥所建设。

加强民主法制建设。自觉接受同级人民代表大会及其常委会的监督，支持人民政协履行政治协商、民主监督、参政议政职能，主动听取各民主党派、工商联、无党派人士以及工会、共青团、妇联等群众团体的意见和建议。加强与人大代表和政协委员的联系，认真办理人大代表建议和政协委员提案。进一步健全基层民主制度，突出抓好社区建设，加强社区自治管理，构建管理有序、文明祥和新型社区。继续开展“四五”普法教育，大力发展法律服务和法律援助事业，不断完善“大调解”工作机制。加强行政执法监督，规范行政执法行为。强化信访工作责任制和责任追究制，及时、有效地处置和化解各种社会矛盾。完善各类突发事件应急处置预案，强化社会治安综合治理，依法打击各种违法犯罪活动，构建长效治安防控体系，建设“平安泰州”。切实抓好安全生产，防止重特大事故发生。

三、加强政府自身建设

适应新形势、新任务、新要求，以十六届四中全会精神为指导，进一步加强政府自身建设，努力建设“为民、务实、清廉”政府。

1、加强能力建设，提升施政水平。把加强学习作为提升能力的重要抓手，深入开展学习型机关、学习型班子、学习型干部创建活动，强化知识培训、能力培训和综合素质培训，引导全体公务员加强学习，博求新知，增长才干。把加强调查研究作为提升能力的重要环节，结合“十一五”规划的编制，深入研究经济社会发展中的重大问题，进一步增强政府工作的系统性和前瞻性。把实践创新作为提升能力的核心，力求在三个方面取得新进步：引导各类经济主体主动适应宏观调控，着力化解经济紧运行中的矛盾和问题，切实加强和改善对企业、项目和投资的服务，优化经济发展软环境，努力在提升服务发展能力上取得新进步；强化对重点经济指标、重点企业发展和重点工程建设进展情况的监测，把握好政策调节的增量导向、科技导向、集约导向和民生导向，坚持财政资金、用地计划、能源供应、信贷投入向重点区域、重点产业倾斜，努力在提升调节经济能力上取得新进步；以“411工程”为总抓手，进一步完善沿江开发、招商引资、全民创业、工业强市、城市建设等重点工作推进机制，健全组织体系，落实工作责任，努力在提升组织推进能力上取得新进步。

2、转变政府职能，改善行政管理。继续抓好行政管理体制改革，完成市、县政府部分机构改革任务；加快经营性服务类事业单位以“三置换一保障”为核心的改制工作，确保完成改革任务；深化行政审批制度改革，扩大集中审批范围，加强市行政服务中心建设。切实转变政府管理职能，在继续加强经济调节和市场监管的同时，更加重视社会管理，建立健全各种应急机制和体系，提高应对突发事件的能力；更加重视公共服务，构建公共财政，完善公共设施，增加公共产品的供给。进一步改进行政管理，完善科学民主决策机制，健全重大问题集体决策制度和专家咨询制度，实行社会公示和社会听证制度；坚持依法行政，严格按照法律规定的权限和程序行使权力、履行职责，做到有权必有责、用权受监督、侵权要赔偿；扩大政务公开，试行网上审批，加快电子政务建设，为群众提供公开、透明、高效服务。

3、大兴求实之风，狠抓工作落实。大力弘扬实干精神，努力形成求实、务实、落实的良好作风。坚持以正确的政绩观抓落实，重实际、鼓实劲，出实招、求实效，使各项工作经得起实践的检验、群众的检验和历史的检验；坚持领导带头抓落实，把主要时间和精力放在重点工作、重大项目和重要活动上，充分发挥领导在抓落实中的核心和表率作用；坚持顶真碰硬抓落实，直面具体矛盾、落实具体措施、解决具体问题，确保政策措施落实到位、工作一抓到底；坚持凝聚合力抓落实，加强部门、地区之间的协调沟通，严肃政务纪律，努力形成政令畅通、协同推进的工作格局；坚持严格考评促落实，强化目标管理，强化效能监察，强化责任追究，确保目标分解到位、压力传递到位、工作落实到位。

4、坚持从严治政，树立清廉形象。认真落实廉政建设责任制，确保横向到边、纵向到底。教育和引导政府机关全体工作人员树立正确的世界观、人生观、价值观和权利观、地位观、利益观，常修为政之德、常思贪欲之害、常怀律己之心，时刻牢记“两个务必”要求，筑牢拒腐防变的思想防线。加强制度建设，用制度规范权力运行、约束公务员从政行为，努力从源头上预防和治理腐败。坚持内部监督与外部监督相结合、行政监督与法律监督相结合、组织监督与群众监督相结合，健全完善权力运行监督体系。加大执纪执法力度，深化纠风和专项治理工作，从严整治政风、行风，树立清正廉洁的政府形象。

各位代表，新的形势催人奋进，新的事业任重道远。让我们紧紧团结在以胡锦涛同志为总书记的党中央周围，高举邓小平理论和“三个代表”重要思想伟大旗帜，牢固树立和认真落实科学发展观，在中共泰州市委的领导下，紧紧依靠全市人民，与时俱进，开拓创新，励精图治，扎实工作，为完成全年各项目标任务，推进全市“五年总量翻番、八年全面小康”进程而不懈奋斗！
